# Harmonization of alcohol use data and mortality across a multi‐national HIV cohort collaboration

**DOI:** 10.1111/acer.15522

**Published:** 2025-01-08

**Authors:** Suzanne M. Ingle, Adam Trickey, Anastasia Lankina, Kathleen A. McGinnis, Amy Justice, Matthias Cavassini, Antonella d’ Arminio Monforte, Ard van Sighem, M. John Gill, Heidi M. Crane, Niels Obel, Inma Jarrin, Elmar Wallner, Jodie Guest, Michael J. Silverberg, Georgia Vourli, Linda Wittkop, Timothy R. Sterling, Derek D. Satre, Greer A. Burkholder, Dominique Costagliola, Jonathan A. C. Sterne

**Affiliations:** ^1^ Population Health Sciences, Bristol Medical School University of Bristol Bristol UK; ^2^ Institute of Immunity and Transplantation UCL London UK; ^3^ US Department of Veteran Affairs West Haven Connecticut USA; ^4^ VA Connecticut Healthcare System West Haven Connecticut USA; ^5^ Department of Internal Medicine Yale University School of Medicine New Haven Connecticut USA; ^6^ Service of Infectious Diseases Lausanne University Hospital and University of Lausanne Lausanne Switzerland; ^7^ ICONA Foundation Milan Italy; ^8^ Stichting HIV Monitoring Amsterdam The Netherlands; ^9^ Division of Infectious Diseases University of Calgary Calgary Alberta Canada; ^10^ Department of Medicine University of Washington Seattle Washington USA; ^11^ Rigshospitalet Copenhagen University Hospital Copenhagen Denmark; ^12^ National Centre of Epidemiology Carlos III Health Institute Madrid Spain; ^13^ CIBER de Enfermedades Infecciosas Instituto de Salud Carlos III Madrid Spain; ^14^ Landeskrankenhaus Graz II, Standort West Graz Austria; ^15^ Department of Epidemiology, Rollins School of Public Health Emory University Atlanta Georgia USA; ^16^ Division of Research Kaiser Permanente Northern California Pleasanton California USA; ^17^ School of Medicine University of Athens Athens Greece; ^18^ Univ. Bordeaux, INSERM, Institut Bergonié, BPH, U1219, CIC‐EC 1401 Bordeaux France; ^19^ INRIA SISTM team Talence France; ^20^ CHU de Bordeaux, Service d'information médicale, INSERM, Institut Bergonié, CIC‐EC 1401 Bordeaux France; ^21^ School of Medicine Vanderbilt University Nashville Tennessee USA; ^22^ Department of Psychiatry and Behavioral Sciences University of California San Francisco California USA; ^23^ Division of Infectious Diseases University of Alabama Birmingham Alabama USA; ^24^ Sorbonne Université, INSERM, Institut Pierre Louis d'Épidémiologie et de Santé Publique (IPLESP) Paris France; ^25^ NIHR Bristol Biomedical Research Centre Bristol UK; ^26^ Health Data Research UK South‐West Bristol UK

**Keywords:** alcohol, AUDIT‐C, epidemiology, harmonization, HIV

## Abstract

**Background:**

Alcohol use is measured in diverse ways across settings. Harmonization of measures is necessary to assess effects of alcohol use in multi‐cohort collaborations, such as studies of people with HIV (PWH).

**Methods:**

Data were combined from 14 HIV cohort studies (nine European, five North American) participating in the Antiretroviral Therapy Cohort Collaboration. We analyzed data on adult PWH with measured alcohol use at any time from 6 months before starting antiretroviral therapy. Five cohorts measured alcohol use with AUDIT‐C and others used cohort‐specific measures. We harmonized alcohol use as grams/day, calculated using country‐level definitions of a standard drink. For Alcohol Use Disorders Identification Test (AUDIT‐C), we used Items 1 (frequency) and 2 (number of drinks on a typical day). Where alcohol was measured in categories, we used the mid‐point to calculate grams/day. We used multivariable Cox models to estimate associations of alcohol use with mortality.

**Results:**

Alcohol use data were available for 83,424 PWH, 22,447 (27%) had AUDIT‐C measures and 60,977 (73%) recorded the number of drinks/units per week/day. Of the sample, 19,150 (23%) were female, 54,006 (65%) had White ethnicity, and median age was 42 years. Median alcohol use was 0.3 g/day (interquartile range [IQR] 0–4.8) and 0 g/day (IQR 0–20) for those with and without AUDIT‐C. There was a J‐shaped relationship between grams/day and mortality, with higher mortality for PWH reporting no alcohol use (adjusted hazard ratio [aHR] 1.46; 95% CI: 1.23–1.72) and heavier (>61.0 g/day) alcohol use (aHR 1.92; 1.41–2.59) compared with 0.1–5.5 g/day among those with AUDIT‐C measures. Associations were similar among those with non‐AUDIT‐C measures.

**Conclusions:**

Grams/day is a useful metric to harmonize diverse measures of alcohol use. Magnitudes of associations of alcohol use with mortality may differ by setting and measurement method. Higher mortality among those with heavier alcohol use strengthens the case for interventions to reduce drinking.

## Graphical abstract

Diverse measures of alcohol use raise methodological issues for combined data analysis in research collaborations using observational cohorts. Grams per day is a potentially viable measure of alcohol use that can be derived from different measures in health records and calculated across cohorts. Despite measurement harmonization, the association of grams per day of alcohol on outcomes may differ across cohorts. However, higher mortality among those with heavier alcohol use strengthens the case for interventions to reduce drinking.

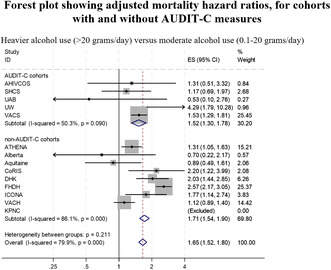

## INTRODUCTION

Excessive alcohol use can lead to deleterious health and social outcomes (WHO, 2018). Alcohol use disorders and alcohol‐related medical diagnoses (e.g., alcoholic liver disease and alcoholic gastritis) are common among people with HIV (PWH) (Williams et al., 2016). Continued alcohol use may exacerbate depression and other mental health problems (Awaworyi Churchill & Farrell, 2017), and may interfere with individuals' efforts to stop smoking, control hypertension, or proactively manage their healthcare (Rittmueller et al., 2015; Thomas et al., 2012). PWH may be particularly susceptible to a range of consequences from alcohol, including hospitalization, physiologic injury, and mortality (Akgun et al., 2013; Bahji et al., 2023; Braithwaite et al., 2014; Jacob et al., 2013; Justice et al., 2016; Korthuis et al., 2012; Lim et al., 2014; Parsons et al., 2014; Trickey et al., 2023; Womack et al., 2013). PWH also face more HIV‐specific harms, for example, alcohol use may decrease adherence to antiretroviral therapy (ART) (Braithwaite & Bryant, 2010), contribute to microbial translocation, and exacerbate chronic inflammation (Monnig et al., 2019). Even low levels of alcohol use are associated with hepatic fibrosis and can aggravate harmful effects of HIV and hepatitis C on the liver (Lim et al., 2014). Alcohol also interacts with many common medications, exacerbating adverse effects (Holton et al., 2017).

To assess alcohol's contribution to morbidity and mortality among PWH, it is important to measure use accurately. Ideally, internationally standardized measures of alcohol use would be consistently collected to inform clinical management. However, the diverse approaches to collection and documentation of alcohol use data hinders the ability to make comparisons across settings or perform meta‐analyses. Research on combining self‐reported measures of alcohol use has reported no consensus on the best method, but of methods available, the quantity‐frequency method (asking about usual frequency and quantity of alcohol use) is reported to be the most reliable and valid (McKenna et al., 2018; Tevik et al., 2021). The Alcohol Use Disorders Identification Test‐Consumption (AUDIT‐C) questionnaire (Bradley et al., 2007; Bush et al., 1998) is used in many settings but its application is far from universal. Several different screening tools have been developed for use in particular populations based on estimates of quantity and frequency of use (Curry et al., 2018). Guidance in the United States recommends using either AUDIT‐C or the National Institute on Alcohol Abuse and Alcoholism (NIAAA) Single Alcohol Screening Question (SASQ) as a screening tool (NIAAA, 2024). In Europe, people are commonly asked about how many drinks they have had in a certain timeframe—an example of the quantity‐frequency method. While the original AUDIT‐C used ≥6 drinks on one occasion as a measure of heavy drinking in addition to usual quantity and frequency of alcohol use, more recent versions of the AUDIT‐C and other instruments use lower cut‐offs for heavy drinking (e.g., ≥4 drinks for women/≥5 drinks for men; Silverberg et al., 2020), or different time frames for calculating frequency.

Given the lack of consensus around recommended methods of measuring alcohol use, we aimed to assess whether data on alcohol use collected by HIV cohort studies using diverse methods could be combined across multiple countries in both Western Europe and North America. We also examined whether associations of alcohol use with mortality varied by measurement method across cohorts.

## METHODS

### Setting

Data were used from the Antiretroviral Therapy Cohort Collaboration (May et al., 2014) (ART‐CC). Established in 2000, ART‐CC combines data from HIV cohort studies across North America and Europe (May et al., 2014). Cohorts comply with their country's regulatory processes. We analyzed data from 14 cohorts collecting alcohol data within the course of usual health care and participating in the ART‐CC's 2019 data update. Cohorts ascertained mortality until the end of 2019, through linkage with vital statistics agencies and hospitals or physician report, and the active follow‐up of participants.

People with HIV eligible for inclusion in the ART‐CC baseline dataset are required to be at least 16 years old and to have had a CD4 count and HIV‐1 RNA viral load (VL) measure within 3 months before and 2 weeks after first starting an ART regimen. The current analyses were restricted to PWH with a measure of alcohol use at any point from 6 months before or at any time during use of ART and who also had data available on all covariates of interest (sex, age, probable mode of HIV acquisition, ethnicity, CD4 count, and VL).

### Measures of alcohol use

Alcohol use data were collected between 1996 and 2019 (start dates varied somewhat by cohort). Five cohorts collected alcohol use data using the AUDIT‐C tool (“AUDIT‐C cohorts”): a three‐item questionnaire which returns a score from 0 to 12 (increasing score means higher risk alcohol use, with a score of 0 indicating no alcohol use in the past year; Bradley et al., 2007). We used the mid‐points of AUDIT‐C Items 1 (frequency of drinking in the past year: Never, Monthly, two to four times per month, two to three times per week, four or more times per week) and 2 (number of drinks on a typical day of drinking in the past year: 1 or 2 drinks, 3 or 4 drinks, 5 or 6 drinks, 7–9 drinks, and 10 or more drinks) to calculate grams of alcohol consumed per day (grams/day). As Item 2 asks about number of drinks, we used country‐specific standard drink sizes to convert this to grams of alcohol. In all countries where AUDIT‐C cohorts were based, the standard drink is equivalent to 14 g of alcohol, except Switzerland where the standard drink is equivalent to 10 g of alcohol (Justice et al., 2016).

Of nine cohorts that did not collect alcohol data using AUDIT‐C, six recorded the number of drinks/units per week/day (see Table S1 for more detail). The remaining three cohorts recorded alcohol data as follows:

- The Italian Cohort of Antiretroviral‐Naïve Patients (ICONA) (standard drink = 14 g) recorded number of glasses of wine/bottles of beer/shots per day (Shanyinde et al., 2019).
- The Canadian Alberta cohort (standard drink = 13.6 g) recorded a categorical variable: None, Level I (<9 drinks per week for women, <14 drinks per week for men), Level II (≥9 drinks per week for women, ≥14 drinks per week for men). We took the mid‐point of the category for Level I and the lowest value for Level II.
- The French Hospital Database on HIV (FHDH) cohort (standard drink = 10 g) recorded a categorical variable: nondrinking, <4 glasses per day, 4–8 glasses per day, >8 glasses per day. For those who drink, we took the mid‐point of the category (where there is a bounded categorization) to define grams of alcohol and took the lowest value for the unbounded category of >8 glasses per day (i.e., nine glasses).

Among AUDIT‐C cohorts, we were able to assess the range of grams/day alcohol for each of the 13 levels of AUDIT‐C score (scores 0–12). Among non‐AUDIT‐C cohorts, values of grams/day were concentrated around 0 and 20 g/day and therefore could not usefully be mapped onto the 13 AUDIT‐C scores. Instead, we grouped the grams/day variable into six categories, matched as closely as possible to groupings of the AUDIT‐C scores (0, 1–3, 4–5, 6–8, 9–10, and 11–12) while also retaining groupings of particular importance, in particular, 0 g/day of alcohol was kept as a separate category to distinguish those with no alcohol use. For meta‐analyses, categories were no alcohol use (0 g/day), moderate alcohol use (0.1–20 g/day), and heavier alcohol use (>20 g/day). Twenty grams per day was chosen as a cut‐off to reflect two standard drinks where a standard drink is 10 g alcohol.

### Covariates

The covariates of interest were sex, age at alcohol measure (16–29, 30–39, 40–49, 50–59, and ≥60 years), probable mode of HIV acquisition (sex between men, injecting drug use [IDU], sex between men and women, Other/unknown), ethnicity (see Appendix S1 for derivation), CD4 count at date of alcohol measure ±3 months (0–49, 50–99, 100–199, 200–349, 350–499, and ≥500 cells/mm^3^), and VL at date of alcohol measure ±3 months (0–3.9, 4–4.9, and ≥5 log_10_ copies per mL).

### Former drinking

In three cohorts from which data on former alcohol use were available, it was possible to separate PWH reporting no current alcohol use into those with former alcohol use and those who were lifetime abstainers. PWH reporting no current alcohol use were categorized as having former alcohol use if they had data on one of the following items: date of stopping harmful drinking, stopping alcohol with or without a withdrawal cure, or whether there was a medical reason for not using alcohol.

### Statistical analysis

Follow‐up began at date of first available alcohol measure and finished at the earliest of: death, loss to follow up, or cohort database close. Information on ascertainment of mortality within cohorts can be found in Table S2. We estimated associations between alcohol use and all‐cause mortality using Cox models adjusted for covariates of interest (sex, age, probable mode of HIV acquisition, ethnicity, CD4 count, and VL) and with baseline hazards stratified by cohort. We also estimated adjusted associations of alcohol use separately for each cohort and then combined estimated hazard ratios using fixed‐effects (inverse‐variance‐weighted) meta‐analysis. Inconsistency in hazard ratios between cohorts was quantified using *I* ^2^ statistics. Results were displayed in forest plots. In the three cohorts with information available, we estimated adjusted mortality hazard ratios comparing PWH with former alcohol use and never alcohol use with moderate alcohol use. To explore the potential effects of age and worse health on alcohol use, we compared those categorized as former drinking to nondrinking/current drinking by age, CD4 cell count, and viral load.

Inclusion of alcohol measures up to 6 months prior to starting ART may lead to the distortion of the association of alcohol use with mortality by subsequent effects of starting ART. We therefore performed a subgroup analysis comparing associations of alcohol use with mortality among those with alcohol measures prior to or on their ART start date with those after ART start date. All statistical analyses were performed using Stata 16.1 (StataCorp, 2019).

## RESULTS

Alcohol data available from the included 14 cohorts are described in Table 1. Five cohorts (three US and two European) collected AUDIT‐C data. Overall, 94,270 (58.0%) patients in the ART‐CC baseline dataset had measures of alcohol use: this proportion varied from 22.3% to 99.5% across cohorts. Cohorts with more complete alcohol recording were more likely to report lower levels of use (data not shown).

**TABLE 1:** Description of cohorts with alcohol data.

	AUDIT‐C data?	Alcohol data available for which years/centres?	*N* in ART‐CC baseline data^a^	*N* of baseline patients with alcohol data	% of baseline patients with alcohol data	*N* with complete data^b^ for analysis
AHIVCOS	Yes	Data only from three centres	1617	1323	81.8	1275
Alberta		2009 onward	1524	1460	95.8	1429
Aquitaine		No known restrictions	2490	2187	87.8	1652
ATHENA		No known restrictions	16,960	6213	36.6	4374
CoRIS		No known restrictions	11,313	5492	48.5	4520
Denmark		No known restrictions	4510	2142	47.5	1847
FHDH		2005 onward	71,610	39,122	54.6	36,469
ICONA		No known restrictions	11,408	8989	78.8	6795
KPNC		July 2013 onward	1524	1220	80.1	1121
SHCS	Yes	2013 onward	6924	6889	99.5	6841
UAB	Yes	2007 onward	1333	976	73.2	927
UW	Yes	2007 onward	1754	1129	64.4	988
VACH		No known restrictions	14,052	3136	22.3	2770
VACS	Yes	2008 onward	15,481	13,992	90.4	12,416
Total			162,500	94,270	58.0	83,424

^a^ Restricted to patients in follow‐up within the years or centres when alcohol data were collected.

^b^ Patients had an alcohol measure in the required time period and had complete data on covariates of interest.

Baseline characteristics of the 83,424 PWH with complete alcohol and covariate data (22,447 and 60,977 from AUDIT‐C and non‐AUDIT‐C cohorts respectively) are shown in Table 2. Most of the PWH in AUDIT‐C cohorts were participants in the Veterans Aging Cohort Study (VACS) cohort of US military veterans and therefore more likely to be male, non‐White, have other/unknown mode of HIV acquisition, be older, and have a higher CD4 and lower VL compared with PWH in non‐AUDIT‐C cohorts.

**TABLE 2:** Characteristics at time of alcohol measure.

Characteristic	All PWH (*N* = 83,424)	AUDIT‐C cohorts (*N* = 22,447)	Non‐AUDIT‐C cohorts (*N* = 60,977)	*p*‐Value
	***N* (%)**			
Female	19,150 (23.0)	2880 (12.8)	16,270 (26.7)	<0.001
Mode of HIV acquisition				
Sex between men	30,655 (36.8)	4792 (21.4)	25,863 (42.4)	<0.001
Injecting drug use	5461 (6.6)	828 (3.7)	4633 (7.6)	
Sex between men and women	31,006 (37.2)	3834 (17.1)	27,172 (44.6)	
Other/unknown	16,302 (19.5)	12,993 (57.9)	3309 (5.4)	
Ethnicity				
White	54,066 (64.8)	11,851 (52.8)	42,215 (69.2)	<0.001
Black	21,605 (25.9)	8290 (36.9)	13,315 (21.8)	
Hispanic	3536 (4.2)	1386 (6.2)	2150 (3.5)	
Asian	795 (1.0)	398 (1.8)	397 (0.7)	
Other	3075 (3.7)	506 (2.3)	2569 (4.2)	
Unknown	349 (0.4)	16 (0.1)	331 (0.5)	
	**Median (IQR)**			
Age (years)	42 (34–51)	48 (39–55)	41 (33–49)	<0.001
CD4 count (cells/mm^3^)	408 (242–610)	441 (265–642)	395 (234–598)	<0.001
Viral load (log_10_ copies/mL)	2.57 (0–4.70)	1.34 (0–4.05)	3.31 (0–4.85)	<0.001
Time from ART start to alcohol measure (days)	148 (−15–1647)	633 (0–2239)	40 (−19–1386)	<0.001

Grams/day increased with increasing AUDIT‐C score, as shown in Table 3. The range of values for grams/day corresponding to each AUDIT‐C score are also shown in Table 3 and show ranges which are overlapping. For example, PWH with AUDIT‐C score 4 used between 0.3 and 17.5 g/day while those with AUDIT‐C score 5 used between 0.3 and 38.4 g/day. Figure S1 presents histograms of grams/day, showing that the proportion of PWH reporting no alcohol use was lower (35% vs. 58%) in AUDIT‐C than non‐AUDIT‐C cohorts. This difference may have been driven by different characteristics of cohort participants: for example, most AUDIT‐C cohort participants were from the VACS cohort and were more likely to have alcohol measured post‐ART than participants in non‐AUDIT‐C cohorts. Figure S2 shows the distribution of grams/day separately for each cohort. Based on these data, we categorized alcohol use as: 0.0, 0.1–5.5, 5.6–13.0, 13.1–28.0, 28.1–61.0, and >61.0 g/day, to maximize agreement between the distributions in AUDIT‐C and non‐AUDIT‐C cohorts.

**TABLE 3:** Grams of alcohol per day corresponding to AUDIT‐C scores.

AUDIT‐C score	*N* (%)	Grams of alcohol per day, median (IQR)	Min, Max
0	7911 (35.2)	0 (0,0)	0, 0
1	4018 (17.9)	0.3 (0.3,0.3)	0.2, 0.3
2	3155 (14.1)	1.5 (1.5,2.1)	0.2, 2.1
3	2459 (11.0)	5.3 (3.5,7.5)	0.2, 7.5
4	1863 (8.3)	11.8 (4.8,16.5)	0.3, 17.5
5	999 (4.5)	12.5 (5.3,17.5)	0.3, 38.4
6	642 (2.9)	12.5 (7.6,17.5)	0.8, 60.3
7	435 (1.9)	17.5 (11.8,27.4)	1.3, 62.7
8	315 (1.4)	27.4 (27.4,38.4)	1.8, 109.7
9	192 (0.9)	43.1 (38.4,60.3)	2.1, 109.7
10	188 (0.8)	60.3 (49.9,60.3)	12.4, 109.7
11	119 (0.5)	87.8 (62.7,87.8)	49.9, 109.7
12	151 (0.7)	109.7 (109.7, 109.7)	78.4, 109.7
Total	22,447 (100)	0.3 (0,4.8)	0, 109.7

Categories of daily alcohol use, together with unadjusted and adjusted mortality hazard ratios (aHR), in cohorts with and without AUDIT‐C measures are shown in Table 4. Non‐AUDIT‐C cohorts had a median of 0 g/day compared with 0.1–5.5 g/day in AUDIT‐C cohorts. For non‐AUDIT‐C cohorts, we could generally only distinguish no‐use, moderate, and heavier alcohol use due to the data collection method used in FHDH, which is the non‐AUDIT‐C cohort contributing the most data. However, the non‐AUDIT‐C cohorts were more likely than AUDIT‐C cohorts to identify PWH with heavier alcohol use, with 8.2% of the sample in the highest two categories compared with 4.3% in the AUDIT‐C cohorts.

**TABLE 4:** Unadjusted and adjusted^a^ mortality hazard ratios (HR) for categorized grams/day of alcohol use, for PWH with and without AUDIT‐C measures.

Grams/day categories	PWH with AUDIT‐C measures (*N* = 22,447)			PWH without AUDIT‐C measures (*N* = 60,977)		
	*N* (%)	HR (95% CI)	aHR (95% CI)	*N* (%)	HR (95% CI)	aHR (95% CI)
0.0	7911 (35.2)	1.98 (1.81–2.16)	1.42 (1.30–1.56)	35,393 (58.0)	0.80 (0.64–0.99)	1.43 (1.13–1.81)
0.1–5.5	9953 (44.3)	1 (reference)	1 (reference)	1876 (3.1)	1 (reference)	1 (reference)
5.6–13.0	2135 (9.5)	1.01 (0.85–1.19)	0.98 (0.82–1.16)	3730 (6.1)	1.09 (0.85–1.41)	1.20 (0.93–1.55)
13.1–28.0	1469 (6.5)	1.53 (1.30–1.80)	1.29 (1.09–1.52)	14,984 (24.6)	0.80 (0.64–1.00)	1.38 (1.09–1.75)
28.1–61.0	677 (3.0)	2.05 (1.67–2.50)	1.46 (1.20–1.79)	3445 (5.7)	1.85 (1.47–2.34)	1.98 (1.56–2.51)
>61.0	302 (1.4)	2.49 (1.90–3.25)	1.86 (1.42–2.44)	1549 (2.5)	3.03 (2.38–3.86)	3.01 (2.35–3.87)

^a^ Adjusted for sex, age, mode, ethnicity, CD4, and viral load. Baseline hazards stratified by cohort.

For the AUDIT‐C cohorts, there was a J‐shaped relationship between alcohol use and mortality, with higher mortality rates for PWH reporting no current alcohol use (aHR 1.42; 95% confidence interval [CI]: 1.30–1.56) and heavier (>61.0 g/day) alcohol use (aHR 1.86; 1.42–2.44) compared with 0.1–5.5 g/day. The adjusted HRs were slightly attenuated compared with the crude HRs. For the non‐AUDIT‐C cohorts, the crude hazard ratio comparing no current alcohol use with 0.1–5.5 g/day was 0.80 (95% CI: 0.64–0.99). After adjustment for covariates, there was a J‐shaped relationship of alcohol use with mortality, with higher mortality for PWH reporting no current alcohol use (aHR 1.43; 1.13–1.81) and heavier (>61.0 g/day) alcohol use (aHR 3.01; 2.35–3.87) compared with 0.1–5.5 g/day.

### Meta‐analysis results

**FIGURE 1:**
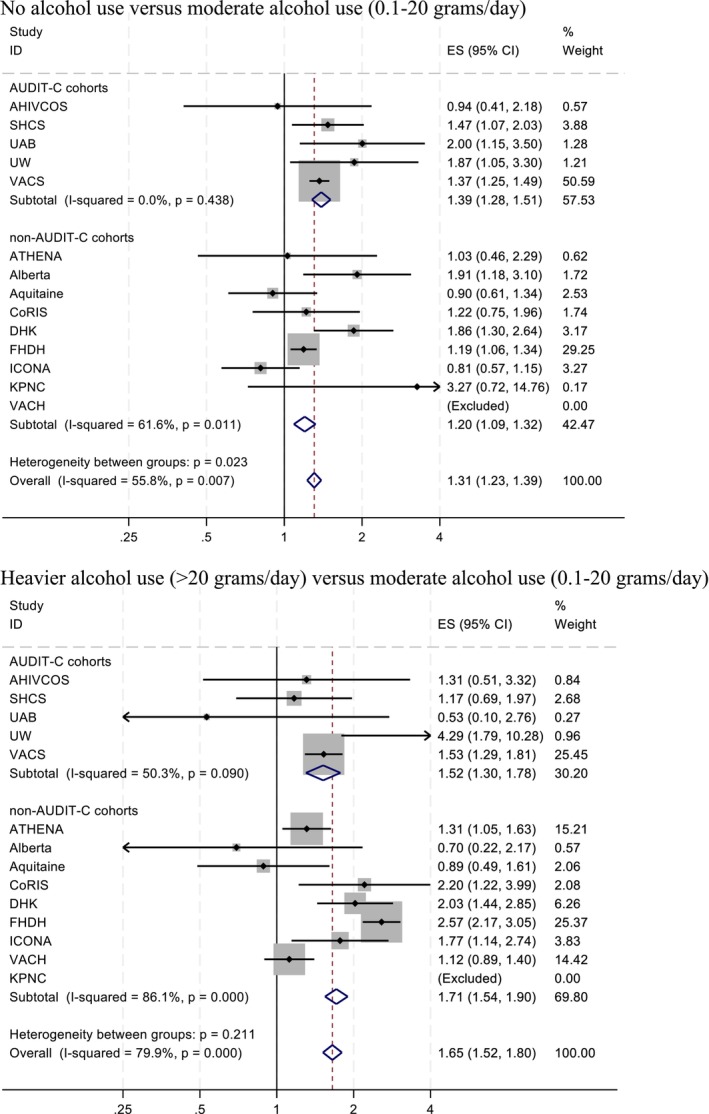
Forest plot showing adjusted mortality hazard ratios, for cohorts with and without AUDIT‐C measures. No alcohol use versus moderate alcohol use (0.1–20 g/day). Heavier alcohol use (>20 g/day) versus moderate alcohol use (0.1–20 g/day). ES, effect size. VACH and KPNC were excluded for having no data on no alcohol use and heavier alcohol use respectively.

Mortality aHRs comparing no current alcohol use (upper panel) and heavier alcohol use (>20 g/day, lower panel) with moderate alcohol use (0.1–20 g/day) across cohorts are shown in Figure 1, together with meta‐analytic summary estimates. The fixed‐effects mortality aHR comparing no use with moderate alcohol use was 1.31 (95% CI: 1.23–1.39), with evidence of between‐cohort variability (*I* ^2^ = 55.8%, heterogeneity *p* = 0.007). The aHRs comparing no use with moderate alcohol use were 1.20 (95% CI: 1.09–1.32; *I* ^2^ = 61.6%, heterogeneity *p* = 0.011) in the non‐AUDIT‐C cohorts and 1.39 (95% CI: 1.28–1.51; *I* ^2^ = 0%, heterogeneity *p* = 0.438) in the AUDIT‐C cohorts. The *p*‐value for differences in the aHRs between AUDIT‐C groups was 0.023.

The fixed‐effects aHR for mortality comparing heavier with moderate alcohol use was 1.65 (95% CI: 1.52–1.80), with more substantial between‐cohort variability than for the comparison of no use with moderate alcohol use (*I* ^2^ = 79.9%, heterogeneity *p* < 0.001). The aHRs comparing heavier use with moderate alcohol use were 1.71 (95% CI: 1.54–1.90; *I* ^2^ = 86.1%, heterogeneity p < 0.001) in the non‐AUDIT‐C cohorts and 1.52 (95% CI: 1.30–1.78; *I* ^2^ = 50.3%, heterogeneity *p* = 0.090) in the AUDIT‐C cohorts. The *P*‐value for differences in the aHRs between AUDIT‐C groups was 0.211.

### Subgroup analyses

Table S3 shows subgroup analyses comparing the aHRs for mortality among those with an alcohol measure prior to their ART start date with those who had an alcohol measure after their ART start date. The association of heavier alcohol use with mortality was greater for post‐ART than pre‐ART measurements. For AUDIT‐C cohorts, the aHRs comparing heavier (>61.0 g) with moderate (0.1–5.5 g) alcohol were 1.48 (95% CI: 0.92–2.39) and 2.28 (1.64–3.16) for pre‐ and post‐ART measurements, respectively. For non‐AUDIT‐C cohorts, the corresponding aHRs were 2.37 (1.58–3.56) and 3.50 (2.54–4.83), respectively. For non‐AUDIT‐C cohorts, there was no elevation in mortality rates for no alcohol use measured pre‐ART (aHR 0.90 (0.61–1.33)).

### Former drinking

Data were available on 3278 PWH from the three cohorts with information on whether not currently using alcohol was due to past alcohol‐associated problems. Of the 1963 (60%) who reported no current alcohol use, 89 (4.5%) reported formerly using alcohol. Among this subsample, those reporting former alcohol use were of a similar age to lifetime abstainers/those who currently use alcohol (median age 42.5 years compared with 41 years). However, they had lower CD4 counts (median CD4 391 cells/mm^3^ compared with 450 cells/mm^3^) and higher VL measures (median 3.25 log_10_ copies/mL compared with 1.60 log_10_ copies/mL). The mortality aHRs compared with moderate alcohol use were 2.23 (95% CI: 1.23–4.04) for those reporting former alcohol use, and 1.01 (0.70–1.46) for those reporting lifetime abstinence.

## DISCUSSION

Our analyses show the feasibility of combining heterogeneous alcohol use measurements and using the resulting harmonized measures in combined analyses. Grams/day is a useful metric to harmonize measures of alcohol use and can be derived with existing common methods of data collection. There was a J‐shaped relationship between grams/day and mortality, with higher mortality for PWH reporting no alcohol use and heavier alcohol use compared with moderate use. However, the association between no alcohol use and mortality needs careful interpretation. When considering this association in a limited subsample of three contributing cohorts, we found elevated mortality risk in people who reported formerly using alcohol compared with moderate alcohol use, and no evidence of a difference in mortality risk for those reporting lifetime abstinence compared with moderate alcohol use. Finally, between‐cohort heterogeneity in effect estimates was lower for cohorts using AUDIT‐C than for cohorts using other measures.

Given the measurement difficulties that exist in this field, existing literature have made efforts to describe the diverse methods of collecting alcohol data and make recommendations. For example, a systematic review in 2021 summarized methods of assessing alcohol use in older populations but found no consensus regarding methods to use (Tevik et al., 2021). Studies exist where both AUDIT‐C and grams per week of alcohol have been measured (Britton et al., 2020), but agreement between measures or effect estimates was not assessed. Therefore, this study is an important addition in the arena of large cohort collaborations where alcohol measures are collected in diverse ways and with measures that cover different aspects of alcohol use. Although converting the different scales to grams/day enabled comparison across cohorts, there remains uncertainty in this conversion due to the use of categories for data collection necessitating the choice of a mid‐point within the category. Care must be taken to understand the different contexts which gave rise to the differing measurements. Settings which collect alcohol data via AUDIT‐C may have different populations than the settings which do not collect AUDIT‐C data. In the present study, most AUDIT‐C cohorts were based in the United States, which may partly explain the differences found. There might also be different modes of data collection across AUDIT‐C and non‐AUDIT‐C cohorts (i.e., face‐to‐face and confidentially collected).

Consistent with the present study, other studies of associations between alcohol use and health outcomes among PWH also found elevated risks of mortality in both those who do not use alcohol and those with heavy alcohol use, compared with low‐to‐moderate alcohol use (Wandeler et al., 2016). This is consistent with studies in people without HIV (Bobak et al., 2016; Di Castelnuovo et al., 2022; Rogers et al., 2013). While self‐reported alcohol measures are useful, they are subject to bias, and efforts to improve detection of alcohol use among patients in clinical settings have included combining self‐reported AUDIT‐C scores with PEth data (Phosphatidylethanol in blood, a direct alcohol biomarker) (Eyawo et al., 2018). This 2018 study found that 15% of those self‐reporting abstinence via AUDIT‐C had a PEth result indicating recent alcohol exposure.

It is hypothesized that the higher mortality rates among those who report no current alcohol use is driven by those who formerly used alcohol who stopped using alcohol for health‐related reasons, including prior alcohol use‐related problems or an alcohol use disorder (Gordon et al., 2020). While full testing of this hypothesis requires information on the reasons why people do not drink (or stop drinking), our study supports this hypothesis, recognizing data were only available from three cohorts, limiting the generalizability of findings. Additionally, we found that former drinkers among these three cohorts had lower CD4 counts and worse viral control, which could contribute to mortality risk. Thus, health factors, including HIV disease severity, rather than any protective effect of light drinking versus nondrinking likely contribute to the J shape findings in our results. Of particular relevance to interpreting these alcohol use patterns and their relationship to health outcomes, a recent systematic review and meta‐analysis found that compared with those who never drank over their lifetime, there was limited evidence of an increased risk of all‐cause mortality among drinkers who drank 25–44 g of alcohol per day and strong evidence of an increased risk for those who drank 45–64 and 65 or more grams per day (Zhao et al., 2023). In the context of this emerging literature and our exploratory subanalysis, we would not interpret our findings to suggest a protective effect of light drinking versus abstinence with regard to mortality risk.

Relatedly, to better understand the population of people who do not use alcohol, the VACS (Gordon et al., 2020) reported in 2019 the results of a survey asking PWH and people without HIV to describe their alcohol use. Those reporting no alcohol use were categorized into three groups: quit after alcohol‐associated problems, quit for other reasons (without alcohol‐associated problems), and lifetime abstainers (LTA). Over half of respondents, both PWH and people without HIV, quit for other reasons. LTAs were distinct from those who quit after alcohol‐associated problems as they had an increased association with the ADH1B polymorphism, protective against harmful alcohol use. However, those who quit after alcohol‐associated problems had better HIV biomarkers suggesting better adherence to ART among this population, highlighting the diversity of the population of people who do not drink.

### Strengths and limitations

The major strength of our study is the large sample size and variety of settings in North America and Europe from which data were derived. Ascertainment of mortality across these varied cohorts is also a major strength (Trickey et al., 2024). This observational study also has limitations. When considering the association between the combined alcohol measure and mortality, we cannot rule out unmeasured or residual confounding. As alcohol data are self‐reported, they may be subject to recall/reporting/social desirability bias: The effect of this may not be evenly spread across drinking categories as PWH who have high alcohol use may be less likely to recall or report how much they drank, compared with PWH who consume no or only small amounts of alcohol. The VACS cohort of military veterans constitutes a large proportion of the data from AUDIT‐C cohorts. This cohort is predominantly older males, which may affect interpretation of AUDIT‐C results in these data. The FHDH cohort constitute a large proportion of the non‐AUDIT‐C cohorts. While FHDH is representative of the French population of PWH, alcohol data are collected in categories from which we take the mid‐point to calculate grams/day, which makes the distribution of grams/day more discretized. Inferences drawn around the effect for former alcohol use among abstainers has limited generalizability due to data being available in only three cohorts.

### Implications

Heterogeneously collected measures of alcohol use can be combined across settings to produce meaningful analyses. We found grams/day of alcohol to be a useful metric that could be derived from all the measures of alcohol use that have been used by the contributing cohort studies (given that, for AUDIT‐C cohorts, the first two items of the AUDIT‐C score are reported and not just the score itself). Nonetheless, the AUDIT‐C score and grams/day measure different, though related, aspects of alcohol use, as illustrated by the overlapping values of grams/day in each AUDIT‐C score category. Unlike the grams/day values, AUDIT‐C scores are calculated dependent on sex‐specific responses which could partly explain the differences in these measures. Higher mortality among those who formerly used alcohol and among those with heavier alcohol use strengthens the case for interventions to reduce drinking and understand risky behaviors. Understanding reasons for stopping drinking is key to interpreting the higher mortality among those with no alcohol use, and so future studies should aim to collect data that could help further examine this association.

## FUNDING INFORMATION

The funders of the study had no role in study design, data collection, data analysis, data interpretation, or writing of this report. The Tripartite COMpAAAS Consortium is funded by 3 grants from the US National Institute on Alcohol Abuse and Alcoholism: U01‐AA026209 to Sterne, J.A.C for ART‐CC.; U01 AA026230 to Satre, D.D and Silverberg, M.J. for KPNC; U01‐AA026224 to Justice, A for VACS. JACS is funded by National Institute for Health Research Senior Investigator award NF‐SI‐0611‐10168. AT is funded by the Wellcome Trust under a Sir Henry Wellcome Postdoctoral Fellowship (222770/Z/21/Z). Funding for the individual ART‐CC cohorts included in this analysis was from Alberta Health, Gilead, ANRS‐MIE (Maladies Infectieuses Emergentes), the French Ministry of Health, the Austrian Agency for Health and Food Safety (AGES), the Dutch Ministry of Health, Welfare and Sport through the Centre for Infectious Disease Control of the National Institute for Public Health and the Environment, the TP‐HIV by the German Centre for Infection Research (DZIF) (NCT02149004), the Instituto de Salud Carlos III through the Red Temática de Investigación Cooperativa en Sida (RD06/006, RD12/0017/0018 and RD16/0002/0006) as part of the Plan Nacional I + D + i and co‐financed by ISCIII‐Subdirección General de Evaluación and the Fondo Europeo de Desarrollo Regional (FEDER), ViiV Healthcare, Preben og Anna Simonsens Fond, Institut National de la Santé et de la Recherche Médicale (INSERM), Bristol‐Myers Squibb, Janssen Pharmaceutica, Merck Sharp and Dohme, the US National Institute on Alcohol Abuse and Alcoholism (U01‐AA026230), the Spanish Ministry of Health, the Swiss National Science Foundation (grant 33CS30_134277), CFAR Network of Integrated Clinical Systems (1R24 AI067039‐1, P30‐AI‐027757, P01 AA029544), the US Department of Veterans Affairs, the US National Institute on Alcohol Abuse and Alcoholism (U01‐AA026224, U01‐AA026209, U24‐AA020794), the VHA Office of Research and Development, and US National Institute of Allergy and Infectious Diseases (Tennessee Center for AIDS Research: P30 AI110527).

## CONFLICT OF INTEREST STATEMENT

MC: Gilead, MSD, and Viiv institution received research grants and expert opinion fees. AS has received grants unrelated to this study and paid to his institution from ECDC. MJG has received honoraria in the last 3 years from ad hoc membership of national HIV advisory boards, Merck, Gilead, and ViiV. HMC has received grants unrelated to this study and paid to her institution from ViiV. IJ has received teaching fees from ViiV, fees for evaluating scientific projects and participating in expert panels from Gilead, and fees for statistical analyses from GESIDA. GAB: Cepheid and Merck Foundation paid research support to my institution. Med‐IQ received honoraria for serving as CME faculty. DC reports an HIV grant from Janssen (2019–2020) and personal fees from Gilead (2020) and Pfizer (2022) for lectures outside the submitted work. EW has recieved grants and honoraria from Gilead, Merck and ViiV. All other authors have nothing to report.

## Supporting information


Appendix S1

## Data Availability

Due to the data sharing agreements between individual cohorts and ART‐CC, the data collected for this study cannot be shared. Data are owned by the individual cohorts and those wishing to access these data should contact the individual cohorts.
