# Supplementary material for: Harmonization of alcohol use data and mortality across a multi‐national HIV cohort collaboration
Source: Alcohol Clin Exp Res (Hoboken). 2025 Jan 8;49(2):407–17. doi: 10.1111/acer.15522 (PMC11828971; doi:10.1111/acer.15522)
Supplement: Supplementary file 1 — Appendix S1 [file ACER-49-407-s001.docx]

**Supplementary material**

**Combining ethnicity and origin variables to create a single ethnicity variable:**

As cohorts had differing data availability relating to ethnicity, to reduce missingness, a new variable was created that also contained information on country of origin as a proxy marker for ethnicity. Where a cohort had data on ethnicity available then this information was used and categorised into groups (White, Black, Asian, Hispanic, Other, Unknown). Where a cohort had no data available on ethnicity, but had data available on origin, then data on origin would be used for this new variable. The ethnicity would be derived as the majority ethnicity of the country of origin (referring to Wikipedia). For countries where there was no majority ethnicity (e.g., Suriname where the two largest ethnic groups are 27% Indian, 22% Maroon-Bushinengue) then the ethnicity would be coded as unknown. Where data on both origin and ethnicity were missing then the ethnicity would be coded as unknown

**Supplementary Table 1:**

|  | **Questions for collecting alcohol data** | **Response** | **Who is asked about alcohol consumption?** |
| --- | --- | --- | --- |
| AHIVCOS | AUDIT-C |  | Data are only available from centers 40, 60 and 80. |
| Alberta | What is your alcohol use in drinks per week? | Level I (<9 drinks/week for women, <14 drinks/week for men)  Level II (≥9 drinks/week for women, ≥14 drinks/week for men)  None  Binge | Alcohol use is/should be asked at every visit from 2009 onwards. |
| Aquitaine | Describe your alcohol consumption? | Previous  Current  Non-drinker  Former drinker |  |
|  | What is your frequency of alcohol consumption? | Occasional  Regular  Acute drunkenness |  |
|  | If regular, number of drinks per day? | Continuous variable, 0+ |  |
| ATHENA | What is the number of units of alcohol you drink per week? | Continuous variable, 0+ | Until 2018 information on alcohol was only collected at the first visit to the hospital following diagnosis. Since 2018 some longitudinal data on alcohol use are available. |
| CoRIS | What is the number of units of alcohol you drink per week? | Continuous variable, 0+ |  |
| Denmark | What is the number of units of alcohol you drink per week? | Continuous variable, 0+ |  |
| FHDH | How many glasses of alcohol do you drinks per day? | 0  <4 glasses  4-8 glasses  >8 glasses | The variables was introduced in 2005. |
| ICONA | Do you drink alcohol? | Yes  No |  |
|  | If yes, how frequently? | Daily  Occasionally |  |
|  | How many glasses of wine per day? | Continuous variable, 0+ |  |
|  | How many bottles of beer per day? | Continuous variable, 0+ |  |
|  | How many shots per day? | Continuous variable, 0+ |  |
|  | Do you abuse alcohol? | Yes  No |  |
| KPNC | What is the average number days per week that you drink alcohol? | Continuous variable, 0+ | July 2013 onwards. NIAAA-based unhealthy alcohol screening questions which assess number of unhealthy drinking days over the prior 90 days, and average weekly quantity and frequency of alcohol use. |
|  | What is the usual number of drinks per day? | Continuous variable, 0+ |  |
| SHCS | AUDIT-C |  | AUDIT-C started in 2013 |
| UAB | AUDIT-C |  | Collected the CNICS PRO assessment at UAB in ~2007 |
| UW | AUDIT-C |  | Collected the CNICS PRO assessment at UW in ~2007 |
| VACH | How many alcoholics drink do you drink per day?  Our references are:  1 Wine 100ml = 9 gr  1 Wine 200ml = 18 gr  1 Beer 200ml = 9 gr  1 Coñac glass = 18 gr  1 Sherry glass 50 ml = 9 gr  1 Cava Glass = 9 gr  1 Vermout glass 50ml = 9 gr  1 Alcoholic Drink (Rum, Martini, ...) = 18 gr  1 Whisky (Or similar) = 27 gr |  |  |
|  | And how often:  < 1 per week  1-2 per week  3-5 per week  > 5 per week  1-2 per month  Only events |  |  |
|  | What is the number of grams of alcohol you drink per day? (Derived from the 2 prior questions) | Continuous variable, 0+ |  |
| VACS | AUDIT-C |  | AUDIT-C collected since 2008 |

**Cohort acronyms:**

AHIVCOS: Austrian HIV Cohort Study, ATHENA: AIDS Therapy Evaluation in the Netherlands, CoRIS: Cohort of the Spanish HIV Research Network, FHDH: French Hospitals HIV Database, ICONA: Italian Cohort Naïve Antiretrovirals, KPNC: Kaiser Permanente Northern California, SHCS: Swiss HIV Cohort Study, UAB: University of Alabama, UW: University of Washington, VACS: Veterans Aging Cohort Study

See here for further information: ART-CC: http://www.bristol.ac.uk/art-cc/whoswho/

**Supplementary Table 2. Methods of ascertaining deaths by cohort**

| **Cohort** | **Methods of ascertaining deaths** |
| --- | --- |
| AHIVCOS | 1. Hospital records 2. Physician 3. Linkage to national death registry |
| Alberta | 1. Hospital records 2. Coroner reports 3. Obituaries 4. Family members/relative 5. Other health care workers such as social workers or pharmacist 6. Annual linkage to provincial death registry |
| Aquitaine | 1. Hospital records 2. Physician 3. Linkage to CEPIDC-INSERM (the Epidemiology Centre on Medical Causes of Death of the French National Institute for Health and Medical Research) after every two years |
| ATHENA | Hospital records |
| CoRIS | 1. Hospital records 2. Linkage to National Death Index (2004-2008) |
| Denmark | Link to the National Death registry |
| FHDH | 1. Hospital records 2. Family members/relatives contacted amongst those lost to follow-up |
| ICONA | 1. Physician 2. Obituaries 3. Linkage to administrative registries |
| KPNC | 1. Various internal sources (administrative, hospital deaths) 2. Linkage to California death certificate files 3. Linkage to Social Security Administration Death Master Files |
| SHCS | Hospital records |
| UAB | 1. Linkage to U.S. SSDI (Social Security Death Index) 2. Family members/relatives 3. Hospital records 4. Obituaries |
| UW | 1. Linkage to Washington State death certificate data until 2017 2. Linkage to U.S. Social Security Death Index until 2014 3. Linkage to National Death Index 4. Family members/relatives 5. Hospital records 6. Obituaries |
| VACH | Hospital records |
| VACS | The VHA Vital Status File (VSF) contains mortality data from multiple VA and non-VA data sources. The production of the VSF began in 2006, and the file was updated quarterly. The population in the VSF was based mainly on the accumulation of social security numbers (SSNs) and demographics (date of birth and gender) found in VHA activity files and used the SSN as the key. The VSF was comprised of two components, the Master File and the Mini File. The Master File contained a record for each combination of SSN, date of birth, and gender found in the source files. Thus, the Master File had more than one record for some SSNs. The VSF Mini File was generated by selecting the ‘best demographics’ for an SSN from possibly inconsistent demographics found on the Master File, and excluded SSNs not associated with Veterans. Dates of death are sourced from the VHA inpatient treatment files, the Social Security Administration’s Death Master File (SSA DMF), Center for Medicare and Medicaid Services (CMS) Medicare enrollment database, and the Beneficiary Identification Records Locator Subsystem (BIRLS). |

**Supplementary Table 3: Unadjusted and adjusted mortality hazard ratios (HR) for alcohol use, stratified by measures recorded pre- and post-ART start**

|  | **Unadjusted Cox models** | | | | | | | |
| --- | --- | --- | --- | --- | --- | --- | --- | --- |
|  | **AUDIT-C cohorts** | | | | **Non AUDIT-C cohorts** | | | |
| **Grams/day categories** | **N (%)** | **Pre-ART HR (95% CI)** | **N (%)** | **Post-ART HR (95% CI)** | **N (%)** | **Pre-ART HR (95% CI)** | **N (%)** | **Post-ART HR (95% CI)** |
| 0.0 | 2149 (33.3) | 1.90 (1.59-2.27) | 5762 (36.0) | 1.63 (1.47-1.81) | 16529 (60.9) | 0.92 (0.62-1.35) | 18864 (58.6) | 1.75 (1.30-2.35) |
| 0.1-5.5 | 2853 (44.3) | 1 (reference) | 7100 (44.4) | 1 (reference) | 829 (3.1) | 1 (reference) | 1047 (3.3) | 1 (reference) |
| 5.6-13.0 | 601 (9.3) | 1.29 (0.94-1.77) | 1534 (9.6) | 1.05 (0.85-1.29) | 1634 (6.4) | 0.81 (0.53-1.25) | 2096 (6.5) | 1.41 (1.02-1.95) |
| 13.1-28.0 | 480 (7.4) | 1.29 (0.93-1.78) | 989 (6.2) | 1.54 (1.27-1.86) | 7298 (26.8) | 0.97 (0.65-1.43) | 7686 (23.9) | 1.67 (1.24-2.25) |
| 28.1-61.0 | 229 (3.6) | 1.89 (1.32-2.71) | 448 (2.8) | 1.89 (1.48-2.41) | 1709 (6.3) | 1.79 (1.22-2.65) | 1736 (5.4) | 2.85 (2.11-3.85) |
| >61.0 | 132 (2.0) | 1.76 (1.10-2.82) | 170 (1.1) | 2.69 (1.95-3.73) | 767 (2.8) | 3.40 (2.26-5.10) | 782 (2.4) | 4.38 (3.18-6.04) |
| **Total** | **6444** |  | **16003** |  | **28766** |  | **32211** |  |
|  | **Adjusted* Cox models** | | | | | | | |
|  | **AUDIT-C cohorts** | | | | **Non AUDIT-C cohorts** | | | |
| **Grams/day categories** | **N (%)** | **Pre-ART aHR (95% CI)** | **N (%)** | **Post-ART aHR (95% CI)** | **N (%)** | **Pre-ART aHR (95% CI)** | **N (%)** | **Post-ART aHR (95% CI)** |
| 0.0 | 2149 (33.3) | 1.50 (1.26-1.80) | 5762 (36.0) | 1.39 (1.25-1.54) | 16529 (60.9) | 0.90 (0.61-1.33) | 18864 (58.6) | 1.80 (1.34-2.41) |
| 0.1-5.5 | 2853 (44.3) | 1 (reference) | 7100 (44.4) | 1 (reference) | 829 (3.1) | 1 (reference) | 1047 (3.3) | 1 (reference) |
| 5.6-13.0 | 601 (9.3) | 1.20 (0.88-1.65) | 1534 (9.6) | 0.93 (0.75-1.14) | 1634 (6.4) | 0.81 (0.53-1.25) | 2096 (6.5) | 1.59 (1.15-2.20) |
| 13.1-28.0 | 480 (7.4) | 1.20 (0.87-1.65) | 989 (6.2) | 1.34 (1.11-1.62) | 7298 (26.8) | 0.96 (0.65-1.42) | 7686 (23.9) | 1.77 (1.31-2.38) |
| 28.1-61.0 | 229 (3.6) | 1.57 (1.09-2.25) | 448 (2.8) | 1.43 (1.12-1.83) | 1709 (6.3) | 1.46 (0.99-2.15) | 1736 (5.4) | 2.44 (1.81-3.30) |
| >61.0 | 132 (2.0) | 1.48 (0.92-2.39) | 170 (1.1) | 2.28 (1.64-3.16) | 767 (2.8) | 2.37 (1.58-3.56) | 782 (2.4) | 3.50 (2.54-4.83) |
| **Total** | **6444** |  | **16003** |  | **28766** |  | **32211** |  |

***** adjusted for sex, age, ethnicity, mode, CD4 and viral load. Baseline hazards stratified by cohort.

**Supplementary Figure 1: Histogram of grams of alcohol per day, for a) AUDIT-C cohorts and b) non AUDIT-C cohorts**

**Supplementary Figure 2: Distribution of grams of alcohol per day, by cohort**
